# Differences in Hyperactivity and Inattention between Adolescents Participating and Non-Participating in A National Polish After-School Athletics Program

**DOI:** 10.3390/jcm8050647

**Published:** 2019-05-10

**Authors:** Dominika Głąbska, Dominika Guzek, Blanka Mellová, Katarzyna Zadka, Katarzyna Żywczyk, Krystyna Gutkowska

**Affiliations:** 1 Department of Dietetics, Faculty of Human Nutrition and Consumer Sciences, Warsaw University of Life Sciences (SGGW-WULS), 159C Nowoursynowska Street, 02-776 Warsaw, Poland; katarzyna_zadka@sggw.pl; 2 Department of Organization and Consumption Economics, Faculty of Human Nutrition and Consumer Sciences, Warsaw University of Life Sciences (SGGW-WULS), 159C Nowoursynowska Street, 02-776 Warsaw, Poland; dominika_guzek@sggw.pl (D.G.); krystyna_gutkowska@sggw.pl (K.G.); 3 Nutrition, Health and Wellness Unit, Nestlé Polska S.A., 32 Domaniewska Street, 02-672 Warsaw, Poland; blanka.mellova@pl.nestle.com (B.M.); katarzyna.zywczyk@pl.nestle.com (K.Ż.)

**Keywords:** hyperactivity, inattention, strengths and difficulties questionnaire (SDQ), attention component, adolescents, #goathletics study

## Abstract

Among the hyperactivity and inattention components, being predictors of the Attention Deficit Hyperactivity Disorder (ADHD) phenotype, there are restlessness, fidgeting, distractibility, lack of reflectiveness and lack of attention components. So far, it was observed that they may be associated with an excessive body mass in children. The aim of the study was to analyze differences of the hyperactivity and inattention between adolescents participating and non-participating in a national Polish after-school athletics program (12–13 years) in a case-control sample. The #goathletics study was conducted among a group of 1014 adolescents—507 representatives for the nationwide physical activity program “Athletics for All” and 507 pair-matched non-participating ones. Assessment of the hyperactivity and inattention was based on a Strengths and Difficulties Questionnaire—Hyperactivity-Inattention subscale (SDQ-HI). It was observed, that in spite of the fact, that the general frequency of hyperactivity and inattention did not differ between groups, the frequency of specific components differed. Especially in the case of girls, for adolescents participating in a national Polish after-school athletics program, the positive attention component was more often observed (39.7%) than for adolescents non-participating (30.0%). It may be concluded, that hyperactivity and inattention components may be less common in the case of active adolescents, than in the case of others.

## 1. Introduction

Hyperactivity and inattention components are predictors of the Attention Deficit Hyperactivity Disorder (ADHD) phenotype as well as minor problems associated with hyperactivity [1]. Though the ADHD may not have been diagnosed, minor hyperactivity may be a cause for problems at school, at home, or during leisure activities as well as with regard to relations with teachers and peers [2] because some studies have indicated a low acceptance of hyperactive children and adolescents [3].

These indicated difficulties include restlessness, fidgeting, distractibility, lack of reflectiveness, and lack of attention components, which may be assessed using a separate Hyperactivity–Inattention subscale of Strengths and Difficulties Questionnaire (SDQ-HI) [4,5]. These components assessed using the SDQ-HI are more often observed in boys as they are, in general, more hyperactive than girls [6,7]. Moreover, the SDQ-HI has a sensitivity of more than 70% in identifying hyperactivity [8].

The association between hyperactivity and inattention component (assessed using the SDQ-HI as well as other scales) and body mass was analyzed in some studies. Erhart et al. [9] conducted a study in a nationally representative group of German adolescents and reported that hyperactive individuals are more likely to be overweight or obese than others, whereas individuals with excessive body mass were more likely to be hyperactive. Moreover, in the study of Egmond-Fröhlich et al. [5] conducted as a part of the KiGGS Study (German Health Interview and Examination Survey for Children and Adolescents), after adjusting for the existing confounders, hyperactivity and inattention (assessed using SDQ-HI) were associated with a higher body mass in adolescent girls, but not in adolescent boys. However, in some other studies, an association between SDQ-HI results and body mass was not found [10,11].

Erhart et al. [9] reported that the mechanism of the association between hyperactivity and inattention component and body mass is unknown, and indicated further studies are necessary to obtain a better understanding of the problem [9]. The main factors in excessive body mass are improper eating behaviors and low physical activity [12], but for the hyperactive individuals, there are some doubts associated with the role of indicated factors, especially for the physical activity. It is stated that the association is probably complex, as children and adolescents with hyperactivity are supposed to have a higher level of physical activity than others, but at the same time, the ADHD phenotype is associated with some typical behaviors which include a lower level of physical activity [9]. It is especially indicated that such children and adolescents may spend more time watching television and playing computer games, that may reduce their leisure time spent actively [13], as well as their parents and caregivers may promote the indicated sedentary activities to reduce their tension [14]. Moreover, as indicated by Cook et al. [14], the only study which analyzed association between physical activity and ADHD phenotype, being the study by Kim et al. [15], proven that in fact for such individuals lower physical activity levels were observed, than for others, and they less often participated in both organized sports and vigorous physical activities. Therefore, physical activity must be treated as a potential factor influencing excessive body mass of hyperactive individuals, but these complex associations should be studied.

The aim of the conducted study was to analyze differences of the hyperactivity and inattention between adolescents participating and non-participating in a national Polish after-school athletics program (12–13 years) in a case-control sample.

## 2. Experimental Section

### 2.1. Ethics Approval Statement

The #goathletics study was conducted according to the guidelines laid down in the Declaration of Helsinki, and all the procedures involving human subjects were approved by the Ethics Committee of the Faculty of Human Nutrition and Consumer Sciences of the Warsaw University of Life Sciences (SGGW-WULS) in Warsaw, Poland (No. 16/2017; 19.06.2017).

### 2.2. Study Participants

The study was conducted in two groups of adolescents—a group of individuals participating in the nationwide physical activity program Athletics for All—Lekkoatletyka Dla Każdego (LDK) and a control group of individuals. The LDK program [http://www.lekkoatletykadlakazdego.pl/], that is conducted in Poland since 2014, is a voluntary, free of charge program for primary and secondary school children and adolescents, organized by the Polish Athletic Association and supported by the Ministry of Sport and Tourism and Nestlé Polska S.A. It includes physical activity education and regular athletic training (3 h a week), as well as additional nutritional education. The #goathletics study was planned to assess the results of the LDK program and to analyze the physical performance, body composition and diet in a group of the LDK program participants in the comparison with the control group.

The study groups were recruited in the group of adolescents aged 12–13 years from all the regions of Poland (central, north, north-west, south-west, south, east), while a geographical breakdown was based on the Polish statistical data. Both boys and girls were included, while the higher share of girls was in accordance with the higher share of girls in the LDK program, as indicated previously [16]. The age group of adolescents older than 12 years was recruited for the study, as it is indicated that until this age the ADHD symptoms are developed [17] and, as a consequence, until this age ADHD is diagnosed in the majority of cases [18].

The first stage was a purposive sampling of schools wherein the LDK program was conducted as well as pair-matched schools (from the same cities) wherein neither the LDK nor any other physical activity program was conducted. School selection was conducted in such a way as to ensure the assumed geographical breakdown and an equal share of schools from big cities and small towns.

The second stage comprised a random choice of study participants from the selected schools. The inclusion criteria were: adolescents aged 12–13; regular participation in the LDK program training for at least 1 year (for LDK group)/no participation in the LDK program, either currently or in the past (for control group); no participation in any other physical activity education or nutritional education program; consent agreement of adolescents for study participation; and consent agreement of parent/legal guardian for the participation of their children in the study. The exclusion criteria were: missing data in the completed questionnaires; a diagnosis of disabled cognitive or motor functions, installed with pacemakers and other stimulators; or a diagnosis of epilepsy.

**Figure 1:**
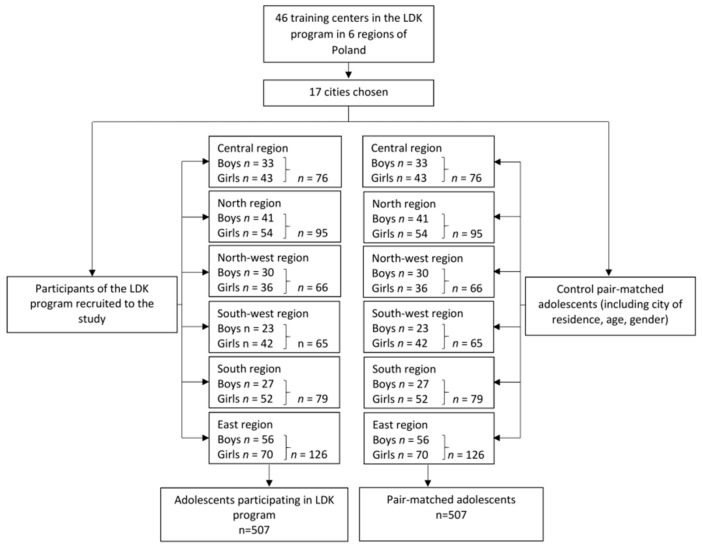
The number of participants in the study from each region of Poland.

The experimental and control groups were pair-matched with regard to the city of residence, age, and gender. When an individual was assigned to the LDK group, s/he was randomly selected from the group of adolescents who participated in the LDK program in a specific school. An individual selected for the control group was pair-matched to an individual from the LDK group, and these subjects were randomly selected from the subgroup of individuals who fulfilled the pair-matching requirements. The number of study participants from each region is presented in Figure 1.

### 2.3. Study Design

The #goathletics study included a comparative analysis of the physical performance, body composition, and diet in a group of participants from the LDK program and the control group. Additional analytical elements included an assessment of contributory factors that influenced the observed associations. Emotional symptoms, conduct problems, hyperactivity and inattention, peer relationships, and prosocial behaviors were identified as the potential contributory/interfering factors [19].

The objective of the presented analysis was to assess differences of hyperactivity and inattention components between adolescents participating and non-participating in a national Polish after-school athletics program.

### 2.4. Hyperactivity and Inattention Assessment

The SDQ is a standardized international instrument, which finds wide application in the assessment of child and adolescent behavior [20]. The questionnaire has five subscales that assess hyperactivity and inattention (namely SDQ-HI subscale) and other components, such as emotional symptoms, conduct problems, peer relationship problems and prosocial behavior [19]. Furthermore, SDQ may be applied as a screening questionnaire for children aged 4–16 [21], filled out by their legal guardians (parents) or teachers; however, adolescents aged 11–16 may fill out the questionnaire by themselves [19].

The hyperactivity and inattention were assessed using the SDQ-HI subscale of the SDQ questionnaire, which is commonly applied to assess mental health outcomes in adolescents practicing sports [22]. The SDQ-HI comprises five questions associated with five components of hyperactivity and inattention: restlessness (described as restless, overactive, cannot stay still for long), fidgeting (constantly squirming), distractibility (easily distracted, concentration wanders), reflectiveness (thinks actions through before acting), and attention (sees tasks through to the end, good attention span). To answer each question, the respondent has to read a sentence presenting the behavior or emotion and is allowed to select one of three options–not true, somewhat true, and certainly true [23].

In this study, subjects aged 12–13 filled out the questionnaire by themselves, as permitted for adolescents aged 11–16 [8]. Their parents neither assisted them nor interrupted their answers; however, the study assistants explained the sentences, if needed, and answered questions that pertained to comprehensibility issues of the participants.

The queries are classified into negative (restlessness, fidgeting, and distractibility) and positive (reflectiveness and attention) ones; for positive queries, the scale was to be reversed. The negative option (“certainly true” for negative components and “not true” for positive ones) was attributed two points; the positive (“not true” for negative components and “certainly true” for positive ones) was assigned 0 points; and the medium option was assigned one point (independently from the character of the feature) [23].

The maximum score on the SDQ-HI is 10 points. For this study, the following cutoff levels were defined: 0–5 points, low needs pertaining to hyperactivity (low hyperactivity and inattention); six points, some needs pertaining to hyperactivity (minor hyperactivity and inattention); and 7–10 points—high needs pertaining to hyperactivity (major hyperactivity and inattention) [21].

In the conducted study, the SDQ-HI subscale was assessed both in its entirety as well as each component separately; the most negative option was defined as a component that was certainly reported, most positive as a component that was not reported, and the medium option as a component that was somewhat reported.

The Cronbach alpha coefficient obtained in the own study for the hyperactivity and inattention was 0.60. It was slightly below the level obtained in the study of Goodman et al. [8], as the Cronbach alpha coefficients were 0.65–0.85 for almost all subclasses, and at the same time, it was higher than obtained by Goodman et al. [8] while the questionnaire was completed by adolescents, as it was 0.44. 

### 2.5. Statistical Analysis

The obtained data are presented as the number and the share of individuals characterized by the reported difficulties/ components of difficulties. Internal reliability of the SDQ-HI was tested using the Cronbach’s alpha coefficient. Differences between groups were identified by using the chi^2^ test. The accepted level of significance was set at *p* ≤ 0.05. Statistical analysis was conducted using Statistica software version 8.0 (StatSoft Inc., Tulsa, OK, USA) and Statgraphics Plus software version 5.1 (Statistical Graphics Corporation, Rockville, MD, USA).

## 3. Results

The frequency of the hyperactivity and inattention did not differ between the compared groups of adolescents, which were characterized by voluntary participation in a physical activity program or the lack of it, while combined results obtained using SDQ-HI scale were assessed (Table 1). Similarly, for a sub-groups of adolescents living in big cities and small towns, there was no association (Supplementary Table S1).

However, while the specific components of the SDQ-HI scale were assessed, there were some differences for the components, but not for restlessness component—neither for the all respondents combined (Table 2) nor for sub-groups of adolescents living in big cities and small towns (Supplementary Table S2).

The frequency of the fidgeting component did not differ between the compared groups, which were characterized by voluntary participation in a physical activity program or the lack of it for the all respondents combined (Table 3). However, in boys from big cities, a difference close to significance was stated (*p* = 0.0812); in adolescents participating in a national Polish after-school athletics program, the fidgeting component was more likely to be not observed (41.9%) than for non-participating ones (28.3%) (Supplementary Table S3). The frequency of the fidgeting component may be interpreted as a higher frequency of this negative component in adolescents non-participating in an athletics program, than in participating ones, which may be attributed to the higher risk of hyperactivity and inattention.

The difference of frequency of the distractibility component between the compared groups, which was characterized by voluntary participation in a physical activity program or the lack of it, was close to a statistical significance for girls (*p* = 0.0599) (Table 4). Those participating in a national Polish after-school athletics program were more likely to be not characterized by this component (44.4%) versus non-participating ones (37.7%). A similar difference was observed for a sub-group of girls from small towns (*p* = 0.0645), as those participating in a national Polish after-school athletics program were more likely to be not characterized by this component (48.2%) versus non-participating ones (35.5%) (Supplementary Table S4). The frequency of the distractibility component may be interpreted as a higher frequency of this negative component in adolescents non-participating in an athletics program, than in participating ones, which may be attributed to the higher risk of hyperactivity and inattention.

The frequency of the reflectiveness component did not differ between the compared groups, which were characterized by voluntary participation in a physical activity program or the lack of it for all the respondents combined (Table 5). However, in boys from big cities, a difference close to significance was stated (*p* = 0.0863); in adolescents participating in a national Polish after-school athletics program, the reflectiveness component was more likely to be observed (40.2%), than for non-participating ones (26.1%) (Supplementary Table S5). The frequency of the reflectiveness component may be interpreted as a lower frequency of this positive component in adolescents non-participating in an athletics program, than in participating ones, which may be attributed to the higher risk of hyperactivity and inattention.

The frequency of the attention component differed between the compared groups of girls (*p* = 0.0213) (Table 6). Those participating in a national Polish after-school athletics program were more likely to be characterized by this component (39.7%) versus non-participating ones (30.0%). A similar difference was observed for a sub-group of girls from big cities (*p* = 0.0261), as those participating in a national Polish after-school athletics program were more likely to be characterized by this component (35.3%) versus non-participating ones (24.5%) (Supplementary Table S6). The frequency of the attention component may be interpreted as a lower frequency of this positive component in adolescents non-participating in an athletics program, than in participating ones, which may be attributed to the higher risk of hyperactivity and inattention.

## 4. Discussion

In the presented own study, in spite of the fact that there were no differences in the combined results obtained using SDQ-HI scale, a number of interesting observations were stated for the specific components. There were some differences for the components, but not for restlessness component. They were stated both for negative components (fidgeting, distractibility) and positive ones (reflectiveness, attention). For all of them, the observed differences may be interpreted as a higher frequency of negative components and a lower frequency of positive components in adolescents non-participating in an athletics program, than in participating ones, which may be attributed to the higher risk of hyperactivity and inattention. The indicated observations may support the role of physical activity and a whole program as a helpful tool to manage hyperactivity and inattention.

Wu et al. [24] concluded that health-promotion programs designed to improve the diet and increase the physical activity of children may mitigate their hyperactivity and inattention and consequently reduce the public health burden of ADHD. The LDK is one such nationwide program that allows children and adolescents from big cities and small towns to participate in physical activity and nutritional education as well as undergo regular athletic training. This free program allows all parents to register their children for the program in their city or town and, thereby, reduces the economic limitations. Furthermore, a volitional choice may be inferred for an individual who participates, as being really involved in physical activity and a healthy lifestyle, whereas an individual who chooses not to participate in this free program in his/her school could be considered to choose a lack of physical activity as participation is not compulsory.

In the mentioned study of Wu et al. [24], the ADHD diagnosis was more common in individuals characterized by a lower quality of diet, lesser level of physical activity, and more screen time playing computer and video games. This observed association between physical activity and hyperactivity may be an outcome of the time spent playing computer and video games because, in general, when an individual spends a lot of time playing computer and video games, s/he does not spend this time on physical activity. Therefore, it must be indicated that the association between hyperactivity and physical activity may result from the association between hyperactivity and playing computer games. In a comprehensive review of the association between internet gaming disorder or pathological video-game use and psychopathology, the authors concluded that one of the strongest associations was observed for hyperactivity, wherein seven of the eight studies included in the review reported full association [25]. However, as the previous study of Erhart et al. [9] indicated an unknown mechanism of association between body mass and hyperactivity, it must be supposed that a rather complex association exists.

Taking into account the previously mentioned results indicating an association between body mass and hyperactivity as well as physical activity and hyperactivity, we undertook more detailed comparisons in this study; we analyzed not only general hyperactivity but also its components. In the present study, the attention component was significantly associated with physical activity; moreover, it needs to be considered that it differed in participating and non-participating in a national Polish after-school athletics. The study of Käll et al. [26] indicated that girls may especially derive higher psychological benefits from extended school-based physical activity. This effect was explained as an outcome of physical activity, which may reduce stress and increase concentration during classes that may have improved their academic proficiency to a greater extent than in the case of boys [23]. This finding is in agreement with the results of the present study because we observed an association between physical activity and the attention component in adolescent girls, but not in adolescent boys.

Furthermore, the attention problems of children and adolescents are indicated to be associated with a sedentary lifestyle and activities such as watching television and playing computer and video games; this was observed in a study by Gentile et al. [27] in a group of children and adolescents aged 8–17, as well in the study of Swing et al. [28] in a group of adolescents and early adults. This may confirm the possible influence of physical activity as a factor that may possibly reduce sedentary behavior and results in less time spent playing computer and video games and, therefore, in a lower level of the attention problem component of the SDQ-HI. However, independently of the mechanism and the fact that the influence of physical activity on hyperactivity may be either direct (physical activity as a positive factor) or indirect (physical activity as a factor that may reduce sedentary behavior being a negative factor), we observed an influence in the present study.

Both in the present study and in the one by Käll et al. [26], the physical activity of the group was analyzed and the social component was considered. In the study of Käll et al. [26], the program organized by the Swedish Sport Confederation included “play and motion” group activities [29]; in the present study, however, athletic training was conducted for the group. The participation of a child or adolescent in group training sessions may influence their emotions and behaviors, both through the activity itself as well as through the forced peer interactions. This results from the fact that, in general, social support may promote better mental health [30]—especially, the group practicing sport may provide social support and interaction [31].

Furthermore, when the association between physical activity and reduced hyperactivity is being analyzed, it must be clarified that the cause and effect relationship was not assessed. In this regard, two potential causal links are possible. On the one hand, in girls, physical activity may promote a higher attention component, reducing the general level of hyperactivity. However, on the other hand, it must be supposed that girls with a higher attention component may be more interested to participate in voluntary physical activity after their regular classes. Despite the fact that, based on the available literature, the first option must be indicated as being possible, the other option should not be rejected and the issue requires further studies for clarification.

Important observations from our study are associated with the fact that, even if the general hyperactivity may not be related to the level of physical activity, an association may be observed for its components, especially for the attention component. The association between physical activity and hyperactivity results from the fact that hyperactivity may be related to a sedentary lifestyle, but also to difficulties in the self-regulation of eating, which together may cause a higher body mass [32].

Moreover, in view of the results of the present study, specific dietary behaviors should be recommended in the prevention of hyperactivity [33] in addition to organized group physical activity, which may be indicated as a potential factor to reduce the risk of hyperactivity, especially in girls. The SDQ is indicated as a screening questionnaire [34] and may allow identification of individuals who would especially benefit from regular organized physical activity as an attempt to reduce the attention problems of children.

In spite of the fact that the study presents a nationwide sample representative for both big cities and small towns in all the regions of Poland, some limitations of the study must be indicated. The assessment of hyperactivity and inattention is challenging, as it is based on own declaration of adolescents, who may want to modify their answers to obtain typical results (as they may be interpreted by adolescent as a better situation). Moreover, the applied intervention, namely LDK program, was associated both with physical activity intervention and nutritional education, which may have contributed to better anthropometric parameters and reduced risk of excessive body mass, as it was observed [35]. However, in such intervention, in spite of the fact that physical activity was the main element, the potential influence of nutritional education may not be neglected. As a result, it should be indicated that the observed situation may be partly attributed to not only physical activity but also a diet. The last, but not least, the presented study was conducted in a specific Polish population, so the observed results should be verified in other countries, in order to verify the potential associations. 

## 5. Conclusions

The components of hyperactivity and inattention, especially the lack of the attention component, may be more common in adolescents with a sedentary lifestyle than in those with active lifestyles. Regular organized group physical activity, accompanied by nutritional education, may be a factor that could reduce difficulties associated with hyperactivity and inattention, especially in girls, both through social support or peer interactions thereby reducing the time spent on sedentary behaviors, but this issue requires further analysis.

## Figures and Tables

**Table 1:** Analysis of the hyperactivity and inattention measured using the Strengths and Difficulties Questionnaire—Hyperactivity-Inattention subscale (SDQ-HI) in groups of adolescents participating and non-participating in a national Polish after-school athletics program.

Gender	Information Obtained on the Basis of the Questionnaire	Adolescents Participating in LDK Program		Adolescents Non-Participating in LDK Program		*p*-Value *
		n	%	n	%	
Boys	Low HI **	172	81.9	163	77.6	0.4230
	Minor HI **	20	9.5	21	10.0	
	Major HI **	18	8.6	26	12.4	
Girls	Low HI **	257	86.5	246	82.8	0.3792
	Minor HI **	25	8.4	35	11.8	
	Major HI **	15	5.1	16	5.4	

* assessed using chi^2^ test; ** HI—Hyperactivity and Inattention.

**Table 2:** Analysis of the restlessness component (negative component) measured using the SDQ-HI scale in groups of adolescents participating and non-participating in a national Polish after-school athletics program.

Gender	Information Obtained on the Basis of the Questionnaire	Adolescents Participating in LDK Program		Adolescents Non-Participating in LDK Program		*p*-Value *
		n	%	n	%	
Boys	Not reported	83	39.5	80	38.1	0.9556
	Somewhat reported	92	43.8	94	44.8	
	Certainly reported	35	16.7	36	17.1	
Girls	Not reported	114	38.4	120	40.4	0.6860
	Somewhat reported	150	50.5	150	50.5	
	Certainly reported	33	11.1	27	9.1	

* assessed using chi^2^ test.

**Table 3:** Analysis of the fidgeting component (negative component) measured using the SDQ-HI scale in groups of adolescents participating and non-participating in a national Polish after-school athletics program.

Gender	Information Obtained on the Basis of the Questionnaire	Adolescents Participating in LDK Program		Adolescents Non-Participating in LDK Program		*p*-Value *
		n	%	n	%	
Boys	Not reported	92	43.8	76	36.2	0.2510
	Somewhat reported	75	35.7	89	42.4	
	Certainly reported	43	20.5	45	21.4	
Girls	Not reported	135	45.5	142	47.8	0.7927
	Somewhat reported	120	40.4	112	37.7	
	Certainly reported	42	14.1	43	14.5	

* assessed using chi^2^ test.

**Table 4:** Analysis of the distractibility component (negative component) measured using the SDQ-HI scale in groups of adolescents participating and non-participating in a national Polish after-school athletics program.

Gender	Information Obtained on the Basis of the Questionnaire	Adolescents Participating in LDK Program		Adolescents Non-Participating in LDK Program		*p*-Value *
		n	%	n	%	
Boys	Not reported	97	46.2	82	39.0	0.1746
	Somewhat reported	86	41.0	89	42.4	
	Certainly reported	27	12.9	39	18.6	
Girls	Not reported	132	44.4	112	37.7	0.0599
	Somewhat reported	137	46.1	140	47.1	
	Certainly reported	28	9.4	45	15.2	

* assessed using chi^2^ test.

**Table 5:** Analysis of the reflectiveness component (positive component) measured using the SDQ-HI scale in groups of adolescents participating and non-participating in a national Polish after-school athletics program.

Gender	Information Obtained on the Basis of the Questionnaire	Adolescents Participating in LDK Program		Adolescents Non-Participating in LDK Program		*p*-Value *
		n	%	n	%	
Boys	Certainly reported	93	44.3	74	35.2	0.1442
	Somewhat reported	96	45.7	115	54.8	
	Not reported	21	10.0	21	10.0	
Girls	Certainly reported	139	46.8	132	44.4	0.2130
	Somewhat reported	151	50.8	150	50.5	
	Not reported	7	2.4	15	5.1	

* assessed using chi^2^ test.

**Table 6:** Analysis of the attention component (positive component) measured using the SDQ-HI scale in groups of adolescents participating and non-participating in a national Polish after-school athletics program.

Gender	Information Obtained on the Basis of the Questionnaire	Adolescents Participating in LDK Program		Adolescents Non-Participating in LDK Program		*p*-Value *
		n	%	n	%	
Boys	Certainly reported	77	36.7	71	33.8	0.8287
	Somewhat reported	113	53.8	118	56.2	
	Not reported	20	9.5	21	10.0	
Girls	Certainly reported	118	39.7	89	30.0	0.0213
	Somewhat reported	159	53.5	176	59.3	
	Not reported	20	6.7	32	10.8	

* assessed using chi^2^ test.

## Supplementary Materials

The following are available online at https://www.mdpi.com/2077-0383/8/5/647/s1, Table S1: Analysis of the hyperactivity and inattention measured using the Strengths and Difficulties Questionnaire—Hyperactivity-Inattention subscale (SDQ-HI) in sub-groups of adolescents living in big cities and small towns, participating and non-participating in a national Polish after-school athletics program, Table S2: Analysis of the restlessness component (negative component) measured using the SDQ-HI in sub-groups of adolescents living in big cities and small towns, participating and non-participating in a national Polish after-school athletics program, Table S3: Analysis of the fidgeting component (negative component) measured using the SDQ-HI in sub-groups of adolescents living in big cities and small towns, participating and non-participating in a national Polish after-school athletics program, Table S4: Analysis of the distractibility component (negative component) measured using the SDQ-HI in sub-groups of adolescents living in big cities and small towns, participating and non-participating in a national Polish after-school athletics program, Table S5: Analysis of the reflectiveness component (positive component) measured using the SDQ-HI in sub-groups of adolescents living in big cities and small towns, participating and non-participating in a national Polish after-school athletics program, Table S6: Analysis of the attention component (positive component) measured using the SDQ-HI in sub-groups of adolescents living in big cities and small towns, participating and non-participating in a national Polish after-school athletics program.
