# Supplementary material for: Differences in Hyperactivity and Inattention between Adolescents Participating and Non-Participating in A National Polish After-School Athletics Program
Source: J Clin Med. 2019 May 10;8(5):647. doi: 10.3390/jcm8050647 (PMC6572158; doi:10.3390/jcm8050647)
Supplement: Supplementary file 1 [file jcm-08-00647-s001.pdf]

Journal of *Clinical Medicine*

MDPI

Article

Differences in hyperactivity and inattention between adolescents participating and non-participating in a national Polish after-school athletics program

Dominika Głąbska, Dominika Guzek, Blanka Mellová, Katarzyna Zadka, Katarzyna Żywczyk and Krystyna Gutkowska

Supplementary Table S1. Analysis of the hyperactivity and inattention measured using the Strengths and Difficulties Questionnaire—Hyperactivity-Inattention subscale (SDQ-HI) in sub-groups of adolescents living in big cities and small towns, participating and non-participating in a national Polish after-school athletics program.

Gender	Location	Information obtained on the basis of the questionnaire	Adolescents participating in LDK program		Adolescents non-participating in LDK program		p-Value*
			n	%	n	%	
Boys	Big cities	Low HI**	91	77.8	63	68.5	0.1458
		Minor HI**	16	13.7	13	14.1	
		Major HI**	10	8.5	16	17.4	
	Small towns	Low HI**	81	87.1	100	84.7	0.7422
		Minor HI**	4	4.3	8	6.8	
		Major HI**	8	8.6	10	8.5	
Girls	Big cities	Low HI**	111	83.5	134	84.3	0.9730
		Minor HI**	11	8.3	13	8.2	
		Major HI**	11	8.3	12	7.5	
	Small towns	Low HI**	146	89.0	112	81.2	0.1320
		Minor HI**	14	8.5	22	15.9	
		Major HI**	4	2.4	4	2.9	

* assessed using chi^2 test; ** HI – Hyperactivity and Inattention.

Supplementary Table S2. Analysis of the restlessness component (negative component) measured using the SDQ-HI in sub-groups of adolescents living in big cities and small towns, participating and non-participating in a national Polish after-school athletics program.

Gender	Location	Information obtained on the basis of the questionnaire	Adolescents participating in LDK program		Adolescents non-participating in LDK program		p-Value*
			n	%	n	%	
Boys	Big cities	Not reported	43	36.8	29	31.5	
		Somewhat reported	52	44.4	49	48.9	0.5621
		Certainly reported	22	18.8	18	19.6	
	Small towns	Not reported	40	43.0	51	43.2	
		Somewhat reported	40	43.0	49	41.5	0.9582
		Certainly reported	13	14.0	18	15.3	
Girls	Big cities	Not reported	57	42.9	67	42.1	
		Somewhat reported	64	48.1	78	49.1	0.9872
		Certainly reported	12	9.0	14	8.8	
	Small towns	Not reported	57	34.8	53	38.4	
		Somewhat reported	86	52.4	72	52.2	0.5952
		Certainly reported	21	12.8	13	9.4	

* assessed using chi^2 test.

Supplementary Table S3. Analysis of the fidgeting component (negative component) measured using the SDQ-HI in sub-groups of adolescents living in big cities and small towns, participating and non-participating in a national Polish after-school athletics program.

Gender	Location	Information obtained on the basis of the questionnaire	Adolescents participating in LDK program		Adolescents non-participating in LDK program		p-Value*
			n	%	N	%	
Boys	Big cities	Not reported	49	41.9	26	28.3	
		Somewhat reported	39	33.3	43	46.7	0.0812
		Certainly reported	29	24.8	23	25.0	
	Small towns	Not reported	43	46.2	50	42.4	
		Somewhat reported	36	38.7	46	39.0	0.7520
		Certainly reported	14	15.1	22	18.6	
Girls	Big cities	Not reported	64	48.1	84	52.8	
		Somewhat reported	46	34.6	55	34.6	0.4941
		Certainly reported	23	17.3	20	12.6	
	Small towns	Not reported	71	43.3	58	42.0	
		Somewhat reported	74	45.1	57	41.3	0.4336
		Certainly reported	19	11.6	23	16.7	

* assessed using chi^2 test.

Supplementary Table S4. Analysis of the distractibility component (negative component) measured using the SDQ-HI in sub-groups of adolescents living in big cities and small towns, participating and non-participating in a national Polish after-school athletics program.

Gender	Location	Information obtained on the basis of the questionnaire	Adolescents participating in LDK program		Adolescents non-participating in LDK program		p-Value*
			n	%	N	%	
Boys	Big cities	Not reported	47	40.2	34	37.0	
		Somewhat reported	55	47.0	36	39.1	0.1080
		Certainly reported	15	12.8	22	23.9	
	Small towns	Not reported	50	53.8	48	40.7	
		Somewhat reported	31	33.3	53	44.9	0.1529
		Certainly reported	12	12.9	17	14.4	
Girls	Big cities	Not reported	53	39.8	63	39.6	
		Somewhat reported	69	51.9	73	45.9	0.2325
		Certainly reported	11	8.3	23	14.5	
	Small towns	Not reported	79	48.2	49	35.5	
		Somewhat reported	68	41.5	67	48.6	0.0645
		Certainly reported	17	10.4	22	15.9	

* assessed using chi[2] test.

Supplementary Table S5. Analysis of the reflectiveness component (positive component) measured using the SDQ-HI in sub-groups of adolescents living in big cities and small towns, participating and non-participating in a national Polish after-school athletics program.

Gender	Location	Information obtained on the basis of the questionnaire	Adolescents participating in LDK program		Adolescents non-participating in LDK program		p-Value*
			n	%	N	%	
Boys	Big cities	Certainly reported	47	40.2	24	26.1	
		Somewhat reported	56	47.9	57	62.0	0.0863
		Not reported	14	12.0	11	12.0	
	Small towns	Certainly reported	46	49.5	50	42.4	
		Somewhat reported	40	43.0	58	49.2	0.5901
		Not reported	7	7.5	10	8.5	
Girls	Big cities	Certainly reported	54	40.6	61	38.4	
		Somewhat reported	74	55.6	89	56.0	0.7264
		Not reported	5	8.3	9	5.7	
	Small towns	Certainly reported	85	51.8	71	51.4	
		Somewhat reported	77	47.0	61	44.2	0.2352
		Not reported	2	1.2	6	4.3	

* assessed using chi[2] test.

Supplementary Table S6. Analysis of the attention component (positive component) measured using the SDQ-HI in sub-groups of adolescents living in big cities and small towns, participating and non-participating in a national Polish after-school athletics program.

Gender	Location	Information obtained on the basis of the questionnaire	Adolescents participating in LDK program		Adolescents non-participating in LDK program		p-Value*
			n	%	N	%	
Boys	Big cities	Certainly reported	40	64.2	22	23.9	
		Somewhat reported	69	59.0	61	66.3	0.2433
		Not reported	8	6.8	9	9.8	
	Small towns	Certainly reported	37	39.8	49	41.5	
		Somewhat reported	44	47.3	57	48.3	0.8224
		Not reported	12	12.9	12	10.2	
Girls	Big cities	Certainly reported	47	35.3	39	24.5	
		Somewhat reported	78	58.6	98	61.6	0.0261
		Not reported	8	6.0	22	13.8	
	Small towns	Certainly reported	71	43.3	50	36.2	
		Somewhat reported	81	49.4	78	56.5	0.4367
		Not reported	12	7.3	10	7.2	

* assessed using chi^2 test.

© 2019 by the authors. Submitted for possible open access publication under the terms and conditions of the Creative Commons Attribution (CC BY) license (http://creativecommons.org/licenses/by/4.0/).
